# Pollution Portrait: The Fourth National-Scale Air Toxics Assessment

**DOI:** 10.1289/ehp.119-a254

**Published:** 2011-06

**Authors:** Bob Weinhold

**Affiliations:** **Bob Weinhold**, MA, has covered environmental health issues for numerous outlets since 1996. He is a member of the Society of Environmental Journalists.

Taken at face value, the results of the fourth edition of the U.S. Environmental Protection Agency’s (EPA) National-Scale Air Toxics Assessment (NATA), released 11 March 2011 are sobering.^1^ Every person in the country is at 10 times or greater risk for getting cancer from outdoor air pollutants than the agency’s general goal of 1 in 1 million,^2^ the average risk is 50 times greater than the goal, and about 5% of the population is at more than 100 times the risk. Almost one-quarter of the population is at increased risk for certain noncancer health effects, primarily respiratory effects.

A closer look reveals the risks might be substantially lower, however. These estimates are based on 2005 emissions, and the EPA says numerous regulations approved and implemented since then have significantly reduced some emissions. The agency also notes that the total cancer toll exacted by the toxics tracked in NATA is very small compared with the risk posed by other known factors. Those combine to create a total risk of 336,000 in 1 million (based on the number of actual cancer cases), with the bulk of the risk widely attributed to lifestyle factors such as tobacco use, diet, lack of exercise, certain types of infections, and radiation.^3^ For additional context, the EPA says radon, which isn’t among the Clean Air Act toxics assessed for NATA, poses a cancer risk of about 2,000 in 1 million.

But Mary Sullivan Douglas, a senior staff associate with the National Association of Clean Air Agencies, cautions that NATA remains incomplete. “There’s a lot that’s not known about the emissions and effects of toxic air pollutants,” she says. “Things could look much worse if we had all the data. We think hazardous air pollutants are very dangerous and need to be addressed, especially for some of the small sources that together form a large part of the NATA inventory.”

## Gaps, Shifts, and New Priorities

NATA addresses 187 outdoor hazardous air pollutants (HAPs) specified in the Clean Air Act^4^ plus diesel particulate matter (PM). One of the major gaps in this iteration of NATA is that it still accounts for only 80 airborne carcinogens and 110 substances linked to noncancer effects. Health risk estimates for 49 of the substances in the toxics list specified by the Clean Air Act haven’t been calculated because of limitations in data, toxicity information, or atmospheric modeling capabilities. The same is true for thousands of other known air toxics.

In addition, states don’t have to meet a consistent national standard for reporting emissions of HAPs. That creates large variations in accuracy, which was a primary problem identified by the EPA’s Science Advisory Board (SAB) a decade ago when it reviewed the first generation of NATA.^5^ There was an attempt to mandate national reporting standards a decade ago, says Thomas Gentile, chief of the Air Toxics Section of the New York State Department of Environmental Conservation and a member of the SAB review panel, but it fell apart, and there are no prospects for such reporting happening any time in the foreseeable future, he says.

There also are problems with the modeling used to estimate outdoor ambient concentrations of each toxic.^6^ According to the EPA, of 69 substances it could evaluate to see how modeled predictions matched with monitored concentrations, less than one-tenth came within 10%, and about 45% had a discrepancy greater than 30%.^7^ Many more of the substances were underpredicted than overpredicted.

NATA also doesn’t cover pollution hot spots, indoor pollutants, dermal or oral exposure routes, or fetal exposures, nor does it consider epigenetic effects.^8^ Moreover, it is most pertinent at the national scale and increasingly less accurate at the state, county, and census tract scales, respectively.

On the other hand, one of the significant improvements in this edition of NATA is the ability to include information on substances that form in the atmosphere from precursor chemicals or are altered by atmospheric reactions, an idea that also was a primary recommendation of the 2001 SAB report.^5^ Only four substances have been addressed: formaldehyde, acrolein, acetaldehyde, and 1,3-butadiene. But the revised risk estimates for these four toxics dramatically altered the findings of this version of NATA—for instance, findings for formaldehyde sent it to the top of the cancer risk list—such that secondary sources are now considered the leading toxicity threat.

Some 83% of ambient formaldehyde, 90% of ambient acetaldehyde, and half of ambient acrolein is formed by secondary reactions, according to an EPA spokeswoman. 1,3-Butadiene is one of the precursors for acrolein. Other precursors for these three toxics include alkenes (for formaldehyde, acetaldehyde, and acrolein), methane and isoprene (for formaldehyde), and alkanes and terpenes (for acetaldehyde). Together, the precursors come from a wide range of natural, manufacturing, combustion, consumer product, and waste stream sources.

Although secondarily formed pollutants may, as a class, be among the leading toxic threats once they are fully accounted, for now many experts consider diesel PM the greatest threat. Noncancer health risks from diesel PM estimated in NATA are one of the eight leading contributors to noncancer effects, but the EPA continues to hold off on making an official estimate for cancer risks. The NATA report states that diesel PM likely poses a cancer threat in the neighborhood of 10–1,000 in 1 million, but the EPA spokeswoman says the agency still doesn’t have enough information to officially estimate the risk, despite decades of study by many experts.

“This is a glaring omission from the NATA risk assessment,” says Robert Sills, toxics unit supervisor in the Air Quality Division of the Michigan Department of Environmental Quality. He recommends the EPA move quickly to finalize its own risk estimate or adopt the one used by California. That stands at 300 in 1 million, according to Michelle Komlenic, an air pollution specialist for the California Air Resources Board. Working with that number, “our highest priority is diesel PM, since it makes the most significant contribution to ambient toxics in California,” Komlenic says.

Despite the lack of an official risk estimate, the agency has moved ahead with various regulations and other programs to reduce diesel engine emissions.^9^ Those types of actions are strongly supported by Richard Kassel, director of the Clean Fuels and Vehicles Project of the Natural Resources Defense Council. “The priority is to accelerate the cleanup of the older diesels and to introduce cleaner diesels. The new diesels are extremely clean compared to the ones they’re replacing,” Kassel says.

With the other information available for the 188 NATA air toxics, the leading identified carcinogens, in generally descending order of magnitude of effect and certainty of linkage, are formaldehyde, benzene, carbon tetrachloride, polycyclic aromatic hydrocarbons, naphthalene, 1,3-butadiene, arsenic compounds, chromium compounds, coke oven emissions, acetaldehyde, acrylonitrile, ethylene oxide, tetrachloroethylene, 1,4-dichlorobenzene, ethylbenzene, nickel compounds, 1,3-dichloropropene, and methylene chloride. For noncancer effects, the leading contributors are acrolein, 2,4-toluene, diisocyanate, chlorine, diesel PM, hexamethylene diisocyanate, hydrochloric acid, and manganese compounds.

The EPA says stationary sources of all types (ranging from power plants, refineries, airports, and large manufacturers to smaller factories, dry cleaners, and gas stations), mobile sources (including both on- and off-road vehicles), and background sources (including natural sources and toxics transported long-range) each contribute about equally to the toxics reported in NATA. All three generate precursors that contribute to secondary pollutants.

The chemical industry plays either a direct or indirect role in many of the dominant chemical source sectors. But Scott Jensen, a spokesman for the American Chemical Council, an industry organization representing chemical manufacturers, declined to comment on NATA, saying his organization doesn’t track this project. Christine Sanchez, spokeswoman for the Society of Chemical Manufacturers and Affiliates, said her organization also hasn’t been following NATA closely, and she too declined to comment.

## Taking Action, Plugging Holes

One of the primary purposes of NATA is to provide general insights about risks posed by the covered toxics so that federal, state, tribal, and local entities can better target their risk reduction efforts. At the federal level, the EPA has several efforts in the works that could contribute to reductions in the toxics covered by NATA. The agency issued a proposed rule^10^ on 16 March 2011 that targets toxics from coal- and oil-fired power plants such as mercury, arsenic, chromium, nickel, hydrogen chloride, and hydrogen fluoride.^11^ It is scheduled to be finalized 16 November 2011.

The EPA has a twice-extended consent decree deadline of 28 July 2011 for issuing a proposed rule^12^ addressing emissions from a wide range of oil and natural gas facilities, and a deadline of 28 February 2012 for issuing a final rule. In November 2011 the agency is scheduled to issue proposed rules for petroleum refinery emissions. Vehicles are another significant source of toxics, and the agency is working on rules that will address a range of emissions, including toxics and greenhouse gases, from light-duty vehicles that become available for model years 2017–2025, with a proposed rule scheduled for release 1 September 2011. The EPA also is working on a risk assessment for coke oven emissions, which the EPA spokeswoman says is scheduled to be completed in phases from 2013 to 2020, although there is no timing for any proposed rules.

The EPA spokeswoman says the agency doesn’t have any plans to develop exposure standards for any of the NATA toxics, as it does for criteria air pollutants such as ozone and sulfur dioxide. Instead, the agency will continue to rely on its efforts, as set out in the Clean Air Act, to address the sources of the toxics emissions, develop mitigation strategies, and rely on modeling and limited monitoring to gauge ambient concentrations and risk.

State officials often appreciate the supplemental information NATA provides their efforts, despite its limitations. “NATA is one of the most promising tools out there for air quality management,” Gentile says. “It allows you to frame a problem more effectively. Is it there yet? No. But it’s getting closer.” For instance, he says it was one of several tools the state used recently in its efforts to reduce high benzene emissions from a point source in western New York.

For Michigan, Sills says, “NATA is very valuable in providing estimates of air toxics ambient air levels and risks across the state. We operate only a limited number of air toxics monitors, for a limited number of parameters, and NATA helps fill the information gaps. [It] also helps us put into perspective concerns expressed by citizens or the press that air toxics hot spots are allegedly causing widespread morbidity or mortality in communities, when ambient air monitoring data are absent.”

California officials voice some support for NATA, but with its own extensive air toxics effort, California Air Resources Board spokesman Dimitri Stanich says his agency is deriving no real benefit from the 2005 data in the new version of NATA. “California is working on 2009 data now,” he says. “We appreciate NATA, but we’ve moved past it.”

That ongoing lag in data that is reported in each updated NATA is a widespread concern. The EPA says it intends to close the gap a little by issuing the next update in 2012 using 2008 data. The EPA spokeswoman says when the next update does come out, it still won’t address indoor exposures, dermal or oral exposure routes, or fetal exposures. But she declined to say which, if any, of the 49 toxics for which there are no risk estimates might be added, which other toxics would be addressed for secondary formation, whether the cancer risk for diesel PM would be finalized, whether any information on dioxins will be included, what new modeling techniques may be used, or what improvements in modeling and monitoring correlation may be feasible.

Sills would like to see much more work done on acrolein and on dioxin and dioxin-like compounds, including monitoring, modeling, and refinement (or adoption, in the case of dioxins) of the risk assessments. Manganese is another critical toxic that needs updating and refinement of its risk assessment, he says. Sills adds that the EPA’s risk assessment benchmarks for acrolein and manganese include uncertainty factors of 1,000, reflecting significant data gaps that should be addressed. “The uncertainties in the exposure assessment are relatively small in comparison to the large uncertainty in the risk assessment benchmarks for these two substances, which makes the significance of benchmark exceedance very unclear,” he explains.

Kassel would like to see the EPA begin to zoom in on some known areas of concern, including near-roadway exposures to associated toxics such as formaldehyde and benzene. “The more granular we can get, the better off we’ll be in allocating scarce resources,” he says.

Such efforts could eventually help federal, state, local, and tribal efforts become more effective in mitigating the wide-ranging adverse health effects of air toxics, which Kassel says is the real bottom line. “I think it’s extremely disturbing that every person in the country faces elevated cancer risk from the air we breathe,” he says. “We live in a country that relies on chemicals, and we’ll never live in a zero-risk world. But much of air pollution is a solvable problem, and we should try to solve it.”

## Figures and Tables

**Figure:**
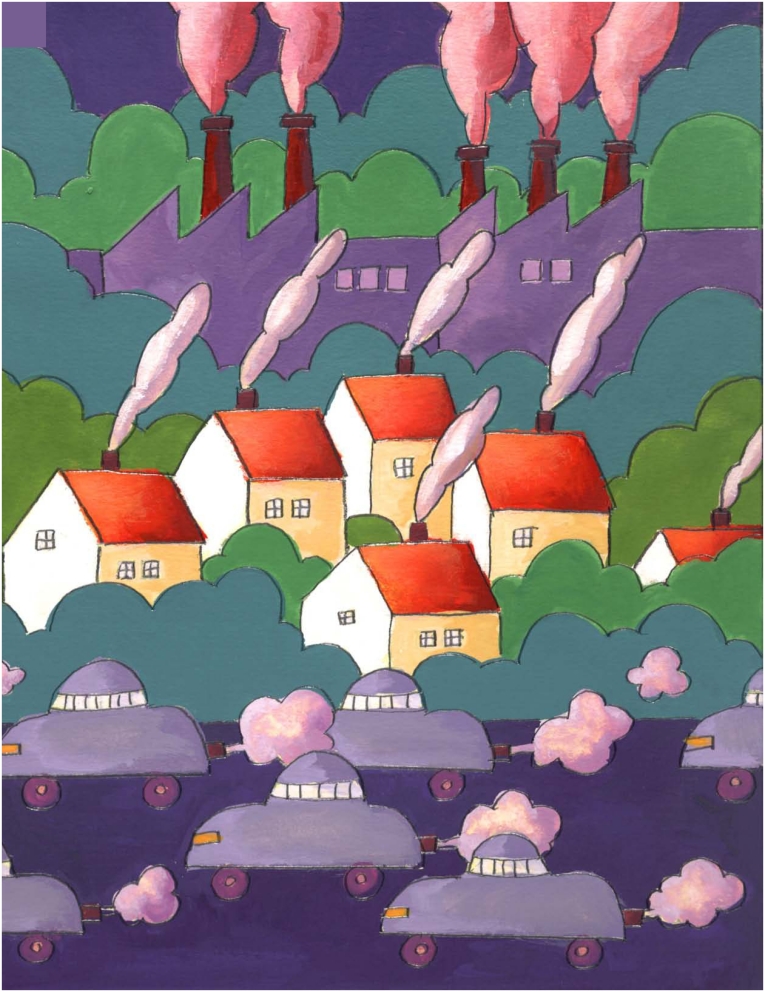


**Figure:**
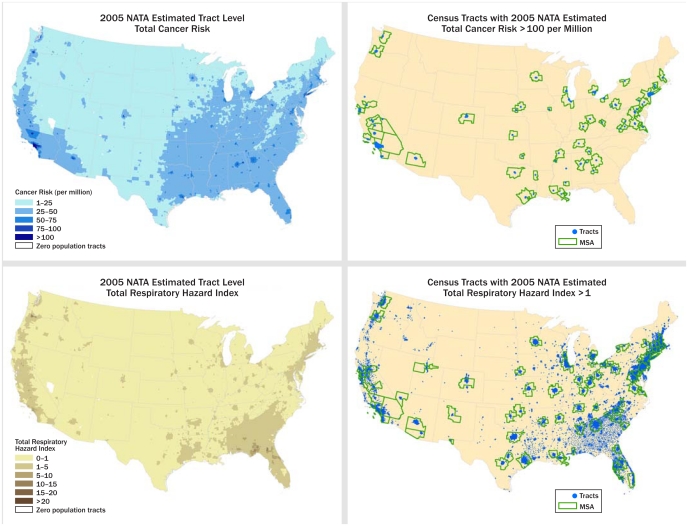
The fourth edition of NATA includes Google Earth maps that show estimated risk levels for each census tract. Individual state maps available at http://tinyurl.com/648h4xv can be downloaded and used to identify the estimated risk averaged across the tract as well as the air toxics contributing to risk levels and their estimated contribution.
